# Severe Normocytic Anemia and Spontaneous Hematomas in Profound Vitamin C Deficiency

**DOI:** 10.7759/cureus.98086

**Published:** 2025-11-29

**Authors:** M Nour Chabalout, Fanny Sotomayor Barrera, Asigul Yimit

**Affiliations:** 1 Internal Medicine, Tower Health Medical Group, Phoenixville, USA

**Keywords:** atypical rash, low socioeconomic status, malnutrition, normocytic anemia, petechiae, scurvy, spontaneous hematoma, vitamin-c

## Abstract

Scurvy is considered rare in developed countries but can occur in patients with a poor diet that lacks vitamin C. Risk factors include, but are not limited to, eating disorders such as anorexia nervosa, social isolation and dietary habits, and limited access to vegetables and produce due to low socioeconomic status. We describe a case of a middle-aged man who presented with bilateral thigh pain and inability to stand up or walk, skin rash, and swelling in his ankles. Initially, he thought he “pinched a nerve” during his daily exercise and that this caused his discomfort in his thighs. He was found to have severe anemia that required blood transfusion, gingival changes, multiple ecchymoses, perifollicular hemorrhage with corkscrew hairs, and an extensive lower extremity rash. His reticulocyte count was low, indicating insufficient bone marrow response. Imaging studies confirmed intramuscular bleeding and spontaneous hematomas. After a thorough investigation and detailed history, it was found that the patient had a very restricted diet due to poor socioeconomic status and neglect. Micronutrient deficiency was suspected, and testing for vitamin C, zinc, folate, and B12 levels was sent. Treatment for scurvy with vitamin C was started as soon as the diagnosis was suspected.

Significant improvement was seen within three days after starting treatment, blood tests confirmed profound vitamin C deficiency, and the patient was eventually discharged to a rehabilitation center. Scurvy remains a challenging diagnosis due to its rarity and its symptoms that may mimic other, more common diseases. This case illustrates the importance of considering uncommon etiologies of severe anemia, especially in patients who have no significant past medical history or clear source of bleeding.

## Introduction

Vitamin C deficiency, characterized by a serum vitamin C level below 0.2 mg/dL, affects millions worldwide, with prevalence varying by age, diet, socioeconomic status, and access to nutritious foods. Those who consume few fruits and vegetables are at increased risk, as humans are unable to synthesize vitamin C and rely on dietary sources. Although scurvy is more common in regions with malnutrition, it occurs globally, with reported prevalence ranging from 7.1% in the United States to 73.9% in northern India [1].

Scurvy results from inadequate vitamin C intake, which is essential for collagen synthesis, a key structural protein maintaining the strength of skin, blood vessels, and connective tissues. Scurvy typically develops after one to three months of severe vitamin C deficiency. Vitamin C acts as a cofactor for proline and lysine hydroxylation, stabilizing collagen and enabling crosslinking. Deficiency leads to unstable collagen, fragile skin and vessels, gingival bleeding, petechiae, and delayed wound healing [2].

Histologically, vitamin C deficiency shows reduced and fragmented collagen fibers, dermal thinning, follicular hyperkeratosis, corkscrew hairs, and perifollicular hemorrhages [2,3]. Gingival tissues demonstrate subepithelial bleeding, capillary dilation, and inflammatory infiltrates [4]. Bone findings include a thin osteoid matrix, subperiosteal hemorrhage, and osteopenia.

In our case, the patient spent months relying on canned food and did not include produce or vitamin C-rich food in his diet. Imaging studies showed that he had intramuscular bleeding. We highlight the importance of keeping a high index for micronutrient deficiencies, which, in many cases, are overlooked and underestimated.

## Case presentation

A middle-aged man presented with progressive fatigue, exertional dyspnea, bilateral thigh swelling, and diffuse skin changes for two weeks. He denied trauma, anticoagulant use, recent infection, or gastrointestinal bleeding.

He reported living alone for four years in poor socioeconomic conditions. His diet consisted of canned food, mainly peanut butter and beans. Over the last six months, he started an exercise program at his home in an attempt to stay physically active and fit. He was lifting weights and doing stretching exercises. He did not consume meals on a daily basis and reported an approximate weight loss of 50-60 pounds over these six months. Collateral history from a family member, who visits him every couple of months, confirmed his poor living conditions. He denied alcohol, tobacco, or illicit drug use.

**Figure 1:**
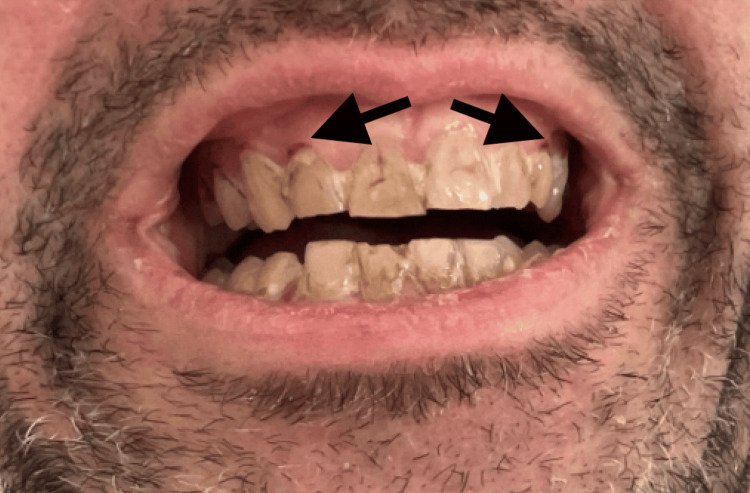
Gingival atrophy with punctuate hemorrhage (arrows)

**Figure 2:**
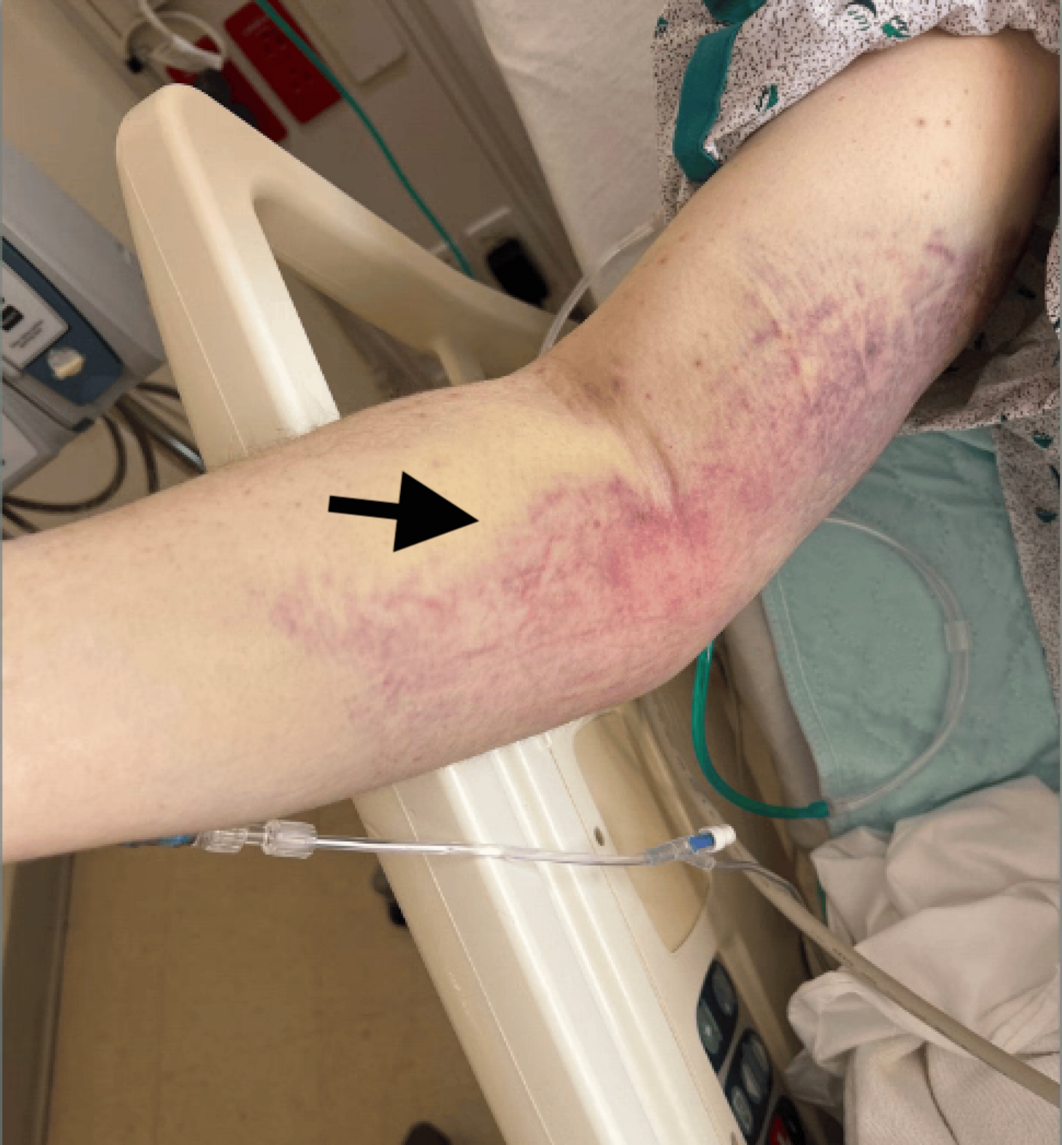
Ecchymosis of the right medial humerus and forearm (arrow)

On admission, he was tachypneic (respiratory rate 33/minute) and tachycardic (heart rate 125 bpm). Physical examination showed marked pallor, poor dentition with gingival atrophy and hemorrhage (Figure 1), bilateral thigh swelling, and diffuse ecchymoses, including over the right humerus and forearm (Figure 2).

**Figure 3:**
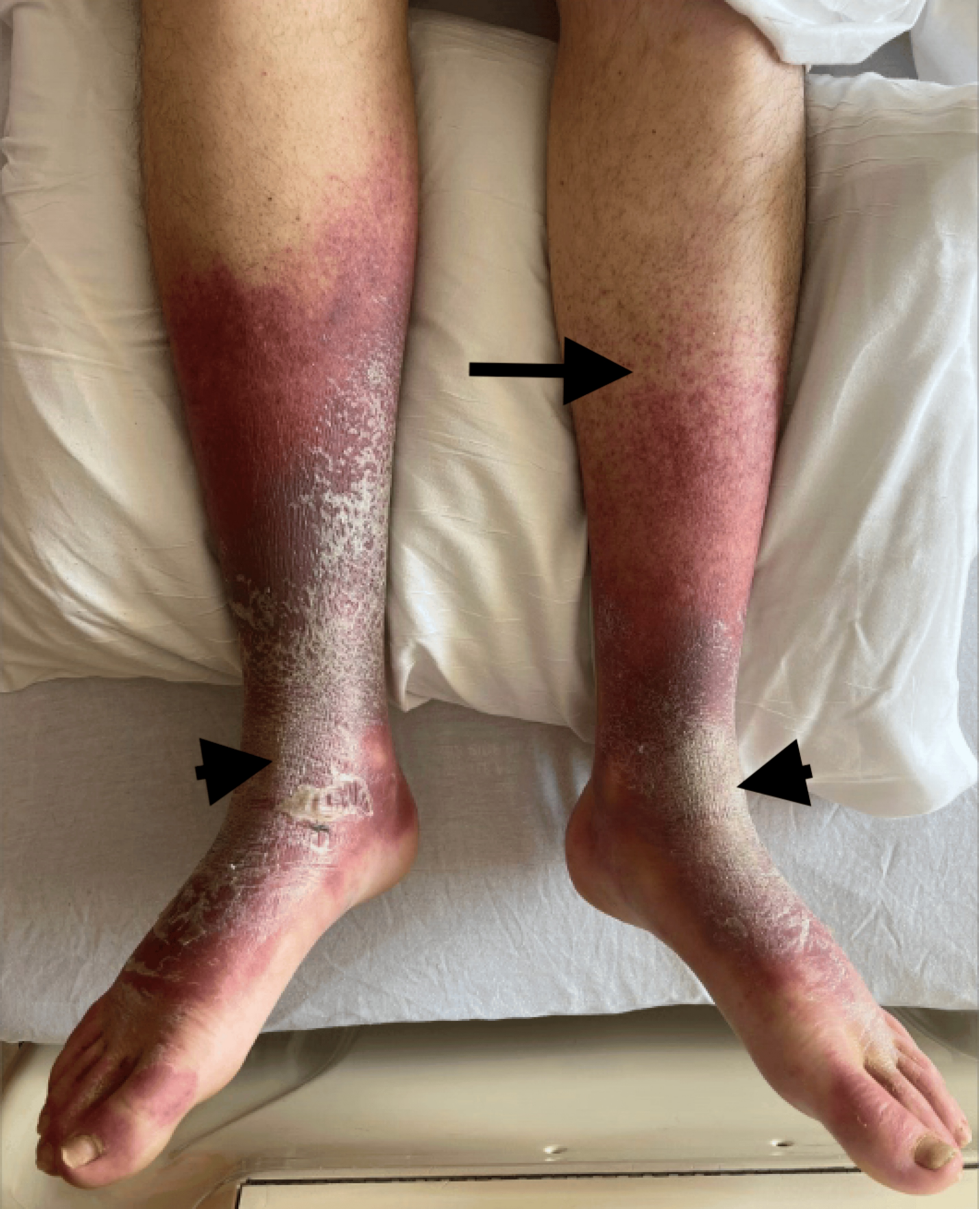
Nonblanching purpura with severe dryness in bilateral lower extremities (arrow) with overlying hyperkeratosis and scaling (arrowheads)

**Figure 4:**
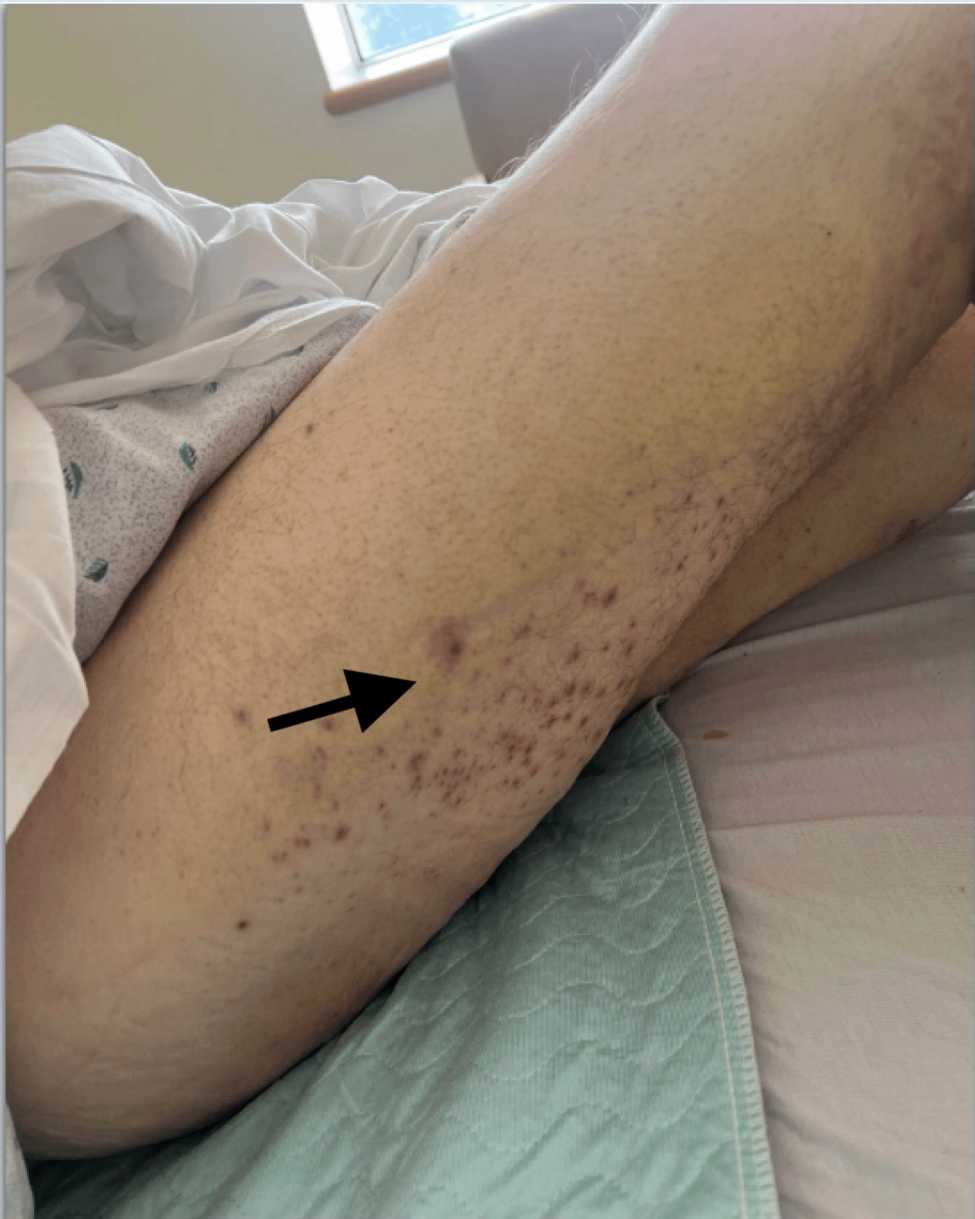
Corkscrew hairs and perifollicular hemorrhage on the lateral and posterior sides of the right thigh (arrow)

Confluent nonblanching purpura with overlying hyperkeratosis extended from both feet to the mid-calves (Figure 3), and perifollicular hemorrhages were evident on the posterior thighs (Figure 4). Both ankles were swollen and tender to palpation, with bedside ultrasound confirming mild joint effusions. No lymphadenopathy or hepatosplenomegaly was noted.

Key laboratory results are summarized in Table 1. A urine drug screen was negative. Peripheral smear showed normocytic anemia with anisopoikilocytosis, helmet cells, and a low reticulocyte count. Hemolysis, marrow failure, and occult gastrointestinal bleeding were excluded with targeted testing. CT of the thighs demonstrated ill-defined intramuscular hematomas; CT of the head, chest, and abdomen was normal.

**Table 1:** Key Laboratory Findings

Test	Result	Reference Range
Hemoglobin	5.6 g/dL	13.5–17.5
Hematocrit	18.4%	41–53
Mean corpuscular volume	88 fL	80–100
Red blood cells	2.1 × 10⁶/µL	4.5–5.9
White blood cells	7.2 × 10³/µL	4.0–10.0
Platelets	256 × 10³/µL	150–400
Reticulocyte count	0.4%	0.5–2.0
Albumin	3.0 g/dL	3.5–5.0
Total protein	5.8 g/dL	6.0–8.3
Lactate dehydrogenase	223 U/L	120–246
Haptoglobin	113 mg/dL	30–200
Total bilirubin	1.8 mg/dL	0.3–1.2
Direct bilirubin	0.4 mg/dL	<0.3
Aspartate aminotransferase	31 U/L	10–40
Alanine aminotransferase	28 U/L	10–55
Alkaline phosphatase	98 U/L	45–115
Creatinine	0.9 mg/dL	0.6–1.3
Blood urea nitrogen	14 mg/dL	7–20
Sodium	138 mmol/L	135–145
Potassium	4.1 mmol/L	3.5–5.0
Bicarbonate	23 mmol/L	22–28
Ferritin	158 ng/mL	30–400
Iron	64 µg/dL	60–170
Total iron binding capacity	310 µg/dL	240–450
Transferrin saturation	21%	20–50
Vitamin B12	321 pg/mL	211–911
Folate	5.8 ng/mL	>5.4
Prothrombin time (PT)	12.4 seconds	11–14
International normalized ratio (INR)	1.0	0.9–1.1
Activated partial thromboplastin time (aPTT)	31 seconds	25–35

The patient received two units of packed red blood cells, improving hemoglobin to 8.2 g/dL. Given the dermatologic findings, nutritional history, and exclusion of alternative etiologies, micronutrient deficiency was suspected, with vitamin C deficiency at the top of the differential diagnoses.

A dietary consult was placed, and the patient was seen by the hospital dietitian on the second day of admission. It was estimated that the patient had lost 10% of his body weight over the last six months with mild depletion of his body fat, indicating malnutrition of moderate degree.

The daily nutritional needs were estimated at 75-90 g of protein, 1500-2000 kcal of calories, and 2000-2200 mL of fluid.

Testing for ascorbic acid level was not available in our hospital, so the test was sent to another hospital, and it took seven days to get the result back. Oral ascorbic acid 500 mg daily was started immediately once the diagnosis was suspected, along with folate, vitamin B12, and the nutritional requirements suggested by the dietitian.

**Figure 5:**
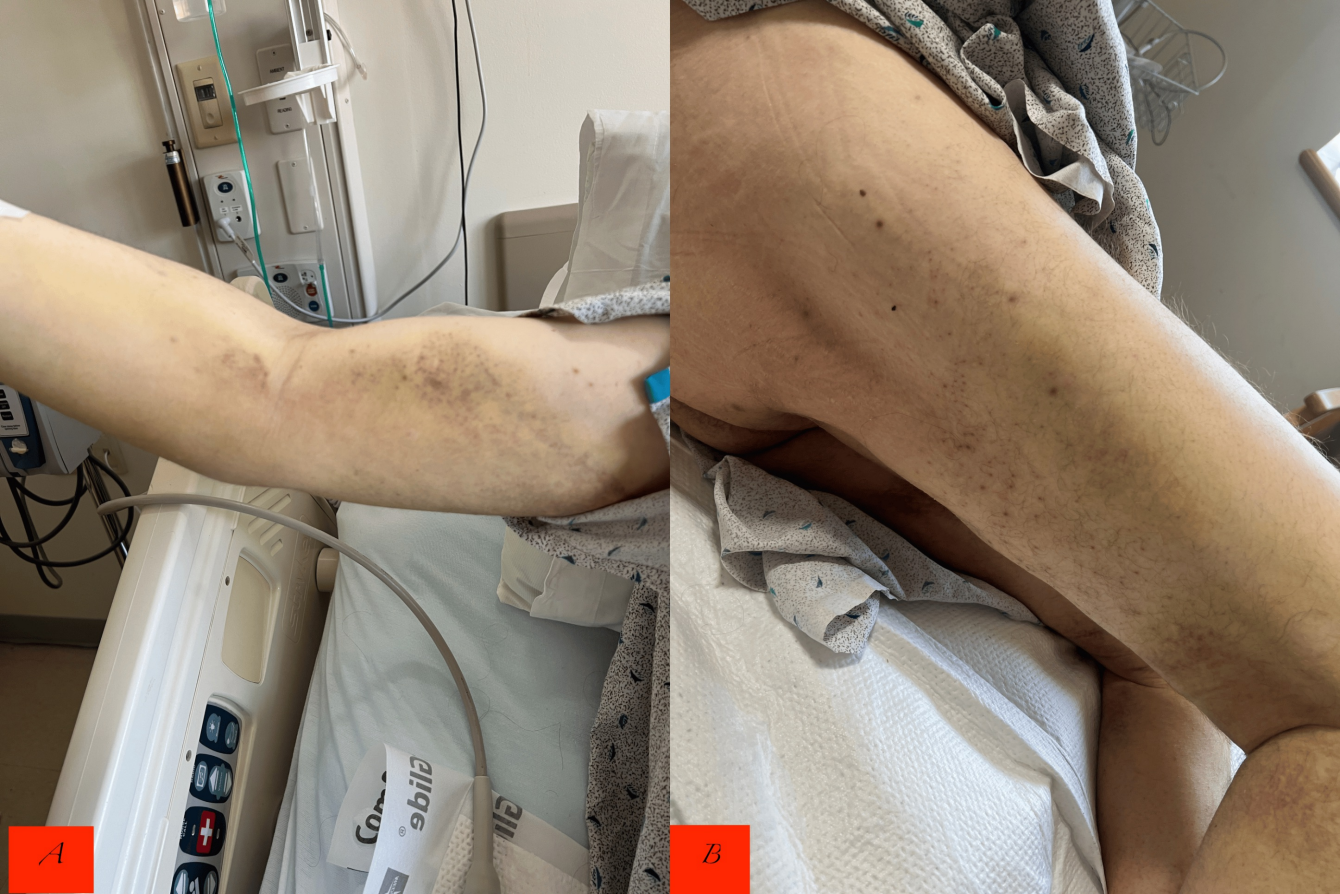
Resolution of the right arm ecchymosis (A) and right thigh perifollicular hemorrhage (B)

Within 72 hours, gingival changes improved, ecchymoses and perifollicular hemorrhages began to resolve (Figure 5), and leg strength increased with physical therapy. Hemoglobin improved to 9.3 g/dL without further transfusion. Ascorbic acid level later returned at <0.1 mg/dL (reference 0.2-2.1 mg/dL), confirming profound deficiency. Workup for autoimmune diseases, including ANA, ANCA, C3, C4, and proteinase 3 antibody, was negative.

The in-hospital social worker was involved, and the patient was provided with a folder containing all resources and food banks he can get free food from. He was later discharged to a rehabilitation facility with additional nutritional counseling and social support.

## Discussion

Scurvy continues to pose a diagnostic challenge because of its rarity, especially in developed countries, and its tendency to mimic other systemic diseases such as vasculitis. Vitamin C plays a vital role in the hydroxylation of proline and lysine during collagen formation, an essential step for maintaining vascular integrity. When deficient, patients develop fragile capillaries, poor wound healing, and reduced iron absorption [5-7].

Anemia associated with vitamin C deficiency has multiple contributing factors, including impaired iron metabolism, chronic inflammation, and bleeding from weakened vasculature [6-9]. In this case, the patient’s normocytic anemia with a low reticulocyte count indicated bone marrow suppression secondary to malnutrition. The presence of spontaneous hematomas and perifollicular hemorrhages signified a severe presentation of scurvy, which has become uncommon in recent times [8,9].

The differential diagnosis initially included vasculitis, coagulation disorders, hemolytic anemia, and bone marrow pathology, all excluded through specific investigations. Diagnosis was ultimately guided by characteristic skin findings, a detailed dietary history, and the patient’s rapid clinical improvement after vitamin C therapy.

This case highlights the necessity of considering micronutrient deficiencies in patients with unexplained anemia or bleeding tendencies, especially those with limited resources or poor nutrition. A thorough history proved crucial; asking detailed questions about dietary habits and, with consent, verifying living conditions with family members helped establish the correct diagnosis.

Early treatment is essential, as administration of vitamin C rapidly restores depleted stores. Recommended doses range from up to 300 mg/day for children to 500-1000 mg/day for adults, continued for one to three months or until complete resolution of symptoms. Alternative adult regimens include 2 g/day for the first three days, followed by 500 mg/day for one week, and then 100 mg/day for one to three months [10].

Long-term management focuses on maintaining a diet rich in fruits and vegetables and addressing underlying causes of malnutrition. Daily vitamin C requirements vary by age, sex, and physiologic state: 15-75 mg for children, 90 mg for men, 75 mg for women, 85 mg for pregnant women, and 120 mg for those who are lactating. Smokers should increase its intake by an additional 35 mg/day due to accelerated depletion of vitamin C [10]. In patients who have limited access to food and nutrition, it is critical to involve a social worker to provide them with the support needed to tackle this issue. Reaching out to our patient’s family members and providing him with places he can get free food from was a pivotal part of our plan to discharge him safely.

## Conclusions

This case illustrates that profound vitamin C deficiency can still occur in individuals living in developed countries, often as a silent manifestation of malnutrition and social isolation. Early recognition and treatment are critical, but sustainable recovery requires addressing the broader determinants of health. A multidisciplinary approach, including physicians, dietitians, and social workers, is vital to assess nutritional needs, ensure access to food, and provide ongoing support.

Clinicians should maintain a high index of suspicion for nutritional deficiencies in patients with limited food access or financial hardship and advocate for systemic interventions that connect vulnerable individuals to nutrition assistance programs, community food resources, and social services. Strengthening these support networks is essential to prevent avoidable morbidity from diseases long thought to be obsolete.
